# Cardiac MR imaging genotoxicity?

**DOI:** 10.1093/eurheartj/ehx719

**Published:** 2017-12-20

**Authors:** Mark A Hill

**Affiliations:** CRUK/MRC Oxford Institute for Radiation Oncology, University of Oxford, Gray Laboratories, ORCRB Roosevelt Drive, Oxford, UK

**This editorial refers to ‘The impact of 1.5 T cardiac magnetic resonance on human circulating leucocytes’^†^, by W.R. Critchley *et al.*, on page 305.**

Magnetic resonance (MR) imaging, including cardiac MR (CMR), is an important, widely used diagnostic tool, and is generally considered a safer alternative to X-ray and radioisotope imaging where there is a clear relationship between exposure to ionizing radiation and increased cancer risk.^1^ MR imaging exposes the patient to a mixture of static magnetic fields, time-varying gradient fields, and pulsed radiofrequency fields, while the exposure of the operator is typically dominated by the static fields. There have been a number of reviews by national and international committees on the potential long-term health risks, and the current consensus is that there is no clear link between fields associated with MR imaging and subsequent health risk.^2^^,^^3^ However, recently there has been increased concern about the potential risks associated with MR imaging due to a limited number of studies reporting an increase in DNA damage following exposure, and this has resulted in calls to limit its use.^4–8^ These and related studies on the potential of MR imaging to result in genotoxicity have also been the subject of a number of recent reviews specifically on genotoxicity.^3^^,^^9^^,^^10^ These also conclude that the ability for MR imaging to produce DNA lesions has yet to be robustly demonstrated, and identify the need for more carefully designed comprehensive studies.

One of the main ways of studying the potential adverse effects associated with MR imaging has been to study DNA damage using γH2AX assays as a measure of DNA double-strand breaks (DSBs). The formation of a DSB results in the phosphorylation of surrounding H2AX histones, forming γH2AX foci. There have been two key *in vivo* studies reporting an increase in DNA damage following exposure using this assay. Fiechter *et al.*^6^ reported a statistically significant enhancement in DSBs (quantified using immunofluorescence microscopy and flow cytometry) in human lymphocytes taken from 20 patients directly following contrast-enhanced 1.5 T CMR, when compared with samples taken prior to imaging (see *Table **1*). Lancellotti *et al.*^5^ reported a significant enhancement in DSBs (quantified using flow cytometry) in T lymphocytes at day 2 [median fluorescence intensity (MFI) = 397 ± 215] and 1 month (MFI = 529 ± 424) post-exposure to unenhanced 1.5 T CMR on 20 healthy men, when compared with samples taken prior to imaging (MFI = 238 ± 88). However, no enhancement was observed at 1 h (MFI = 275 ± 114) and 2 h (MFI = 282 ± 155) post-exposure.

**Table 1:** Comparison of the results from three *in vivo* studies comparing the γH2AX response immediately before and after exposure of patents to 1.5 T CMR, with the response assessed using immunofluorescence microscopy or flow cytometry

*In vivo* study	Patients	Microscopy (foci per cell)		Flow cytometry (MFI)	
		Pre-CMR	Post-CMR	Pre-CMR	Post-CMR
Fiecheter *et al.*^6^	20	0.143 ± 0.191	0.270 ± 0.227	2989 ± 850	3395 ± 906
Brand *et al.*^11^	45	0.116 ± 0.019	0.117 ± 0.019		
Critchley *et al.*^12^	64				
T cells				8680 ± 3090	8410 ± 2730
Monocytes				3470 ±.1350	3340 ± 990

The more recent γH2AX studies of Brand *et al.*^11^ (45 patients) and Critchley *et al.*^12^ (64 patients), the latter found in this issue of the journal, did not show an enhancement 5 min post-1.5 T contrast-enhanced CMR *in vivo* patient exposure (see *Table **1*). Interestingly, the mean number of foci per cell observed by Brand *et al.*^11^ using immunofluorescence microscopy is significantly smaller, and more consistent, than the corresponding data of Fiechter *et al.*,^6^ varying from 0.09 to 0.17 (compared with pre-exposure values ranging from 0 to 0.661 for Fiechter *et al.*). These values are consistent with typical background levels of < 0.2 foci per lymphocyte reported for ionizing radiation biodosimetry studies. While significant differences (both positive and negative) were seen by Fiecheter *et al.* when directly comparing pre-exposure and post-exposure response of individuals, the mean excess foci observed by Brand *et al.* was 0.001 ± 0.001 DSBs per cell.

The methodology used by Critchley *et al.*^12^ is similar to that used by Fiechter *et al.*,^6^ with blood drawn before and after MR imaging of patients, quantified using flow cytometry, and analysed in a blinded fashion. This study was carried out on 64 consecutive consenting patients and this constitutes the largest study to date. All patients received 0.1 mmol/kg Gadovist and underwent a standard ‘viability-type’ clinical CMR using a 1.5 T scanner taking 42 ± 11 min (compared with 68 ± 22 min for Fiechter *et al.* and 30–60 min for Brand *et al.*). CMR was found not to be associated with a significant change of expression in both T cells and monocytes (see *Table **1*); however, there were significant inter-patient variations in expression, with both large increases and decreases observed following CMR. *In vitro* studies were also performed on blood samples from 22 healthy volunteers. Again no difference in γH2AX expression was observed in T cells or monocytes, between blood receiving CMR exposure and unexposed samples left on the bench while the CMR exposures were performed. Interestingly these samples did show a significant increase in γH2AX expression compared with baseline control samples which were processed and analysed immediately. This highlights how sample-handling can affect the observations and the need to treat, process, and analyse the control and exposed samples in an identical fashion, at the same time. The authors note that like the other studies, they did not include all the appropriate control data that are required for more detailed comprehensive studies.

A related study by Fatahi *et al.*^13^ investigated the effect on 11 healthy individuals following repeated exposure to 7 T and 3 T MR imaging, with blood taken between 1 and 4 weeks after the last *in vivo* 7 T MR imaging exposure and γH2AX expression assessed using immunofluorescence microscopy. The mean number of foci per cell observed in these 11 exposed individual was 0.10 ± 0.01, which was not significantly different from the response of 0.09 ± 0.02 observed in 11 unexposed control individuals. Additionally, no significant enhancement was seen in cells exposed *in vitro* 1, 20, and 72 h post-7 T MR imaging.

Accurate quantification of the γH2AX assays is not easy and is sensitive to the preparation, imaging, and scoring criteria. As a result, significant variation can be seen, not only between laboratories but also between individuals. The technique and associated scoring therefore need to be optimized and benchmarked to ensure consistency. Although many cells may be analysed quickly using the flow cytometry technique, its sensitivity is typically lower than can be achieved by scoring individual foci. Care must also be taken when quantifing DSBs, as γH2AX is not exclusive to sites of DSBs, but can also be produced at the site of stall replication forks and in some cases generated as a result of transcriptional activity. When assessing the potential long-term effects of γH2AX and the implied DSB yield, it must also be noted that not all DSBs are equal in terms of their biological efficiency, especially with respect to ionizing radiation. Ionizing radiation, such as X-rays, has a unique ability to produce correlated damage along the path of the electrons produced, and therefore is effective at producing clustered DNA damage, including DSBs that are complex (by virtue of additional base damage or strand breaks within a few base pairs of the DSB). These complex DSBs are more difficult for the cell to repair and, as a result, are significantly more efficient at leading to a range of biological effects than simple DSBs.^9^ Since the fields associated with MR imaging are non-ionizing, they are unable to produce free electrons with enough energy to cause these types of clustered DNA lesions. Therefore, if MR imaging were capable of producing an enhancement in γH2AX, it is important not to compare yields with that produced by ionizing radiation, in order to infer cancer risk. In addition to these γH2AX studies, a range of other biological endpoints, such as the induction of micronuclei and chromosome aberrations, have also been studied, again with contrasting results and interpretations.^3^^,^^9^^,^^10^

**Figure 1:**
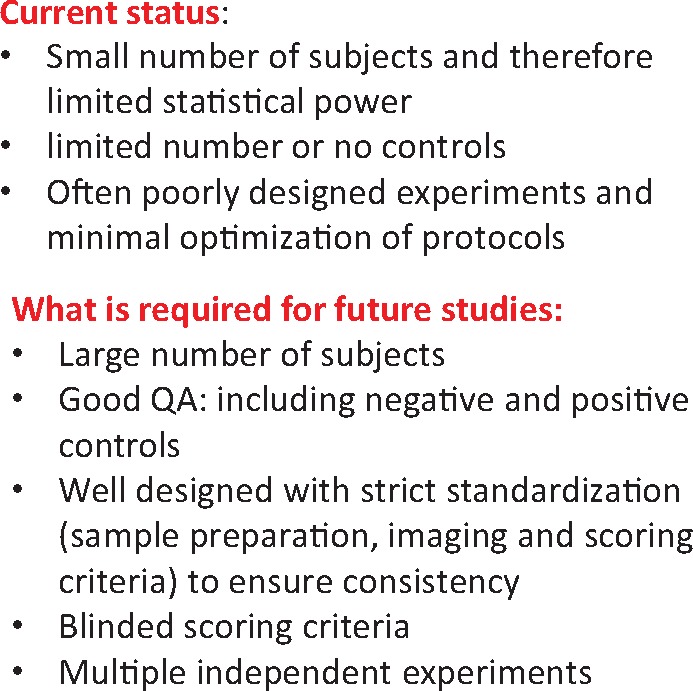
This figure illustrates the current status of MR imaging genotoxicity studies and what is required for future studies.

To date there have been only a limited number of studies published, typically with a limited number of or no controls and often with only a small number of subjects and therefore have limited statistical power. It is essential that any future studies are well designed with strict standardization in experimental design and blinded scoring criteria (*Figure 1*). In addition, a range of quality control measures, including both negative and positive controls (potential ionizing radiation), should be used to benchmark the assays and confirm the validity and reproducibility of the results from multiple independent experiments. It would also be useful to investigate a range of times post-exposure. While there is a tendency to focus on the initial induction of DNA damage, it should be noted that these do not always result in long-term health effects, and it is more relevant to study downstream effects, for example chromosome aberrations for which there is some evidence that this could be a marker of cancer risk. The field could benefit from employing the strict methodology used for biodosimetry of ionizing radiation exposures at national centres.

In summary, the ability for MR imaging to generate DNA lesions has yet to be robustly demonstrated and potential mechanisms are unclear. Even if it was capable of producing damage, then it is essential to avoid overinterpretation of the long-term health risks, especially in light of the ∼50 000 lesions produced daily by endogenous processes and that the fields are non-ionizing and therefore unlikely to produce the complex DNA damage associated with ionizing radiation.
